# Crown Company Beaten on Bridge Suit

**Published:** 1896-04

**Authors:** 


### CROWN COMPANY BEATEN ON BRIDGE SUIT.

The following telegram was received from Dr. Crouse as we were ready for press. Its importance will be appreciated.

"Chicago, Ill., March 23, 1896.

"Dr. James Truman:

"Have beaten Crown Company on bridge suit.

"J. N. Crouse."

